# Double Vagina

**Published:** 1870-08

**Authors:** J. C. Davis

**Affiliations:** Fort Atkinson, Jefferson County, Wis.


ART. IX.—*Double Vagina.* By J. C. DAVIS, M.D., Fort Atkinson, Jefferson county, Wis.

Miss E., of Rock county, in this State, aged twenty-one years, of medium size, and apparently of a good constitution, called at my office to consult me in regard to her health, which she stated had been failing for the past two years, in consequence of having taken cold during menstruation, which brought on prolapsus uteri, as she had been informed. But upon examination per vaginam, I found it to be retroversion of the uterus; and when attempting to replace the same, I discovered a septum commencing at the mesial line, (in a line from right to left,) attached to meatus urinarius, and extending laterally and posteriorly two-thirds of the way to the fourchette, and within the vulva and hymen.

The external genital organs are well developed. The mons veneris well covered with hair. The labia majora and minora are perfect

Its internal attachment commenced within that of vagina to uterus, and extended half around to anterior and posterior mesial line, thence by its edges to anterior and posterior vaginal walls, leaving the os uteri in the right vagina, (or vagina proper,) which is of normal size, shape and capacity.

